# Cross-Linguistic Trends in Speech Errors: An Analysis of Sub-Lexical Errors in Cantonese

**DOI:** 10.1177/00238309211071045

**Published:** 2022-02-08

**Authors:** John Alderete

**Affiliations:** Simon Fraser University, Canada

**Keywords:** Speech errors, phonological encoding, language production, linguistic diversity, Cantonese, syllables, mora, similarity

## Abstract

Though past research on the sound structure of speech errors has contributed greatly to our understanding of phonological encoding, most of this research comes from a small set of majority languages with similar linguistic structures. To increase the linguistic diversity of relevant evidence, a large collection of speech errors was investigated in Cantonese, an under-studied language with unique phonological structures. In particular, the Cantonese data were examined for nine psycholinguistic effects commonly used as a lens on word-form encoding. Detailed quantitative analysis found that Cantonese has eight of these effects, providing broader cross-linguistic support for models based on these patterns. Yet Cantonese also exhibited differences with well-known Indo-European languages by having a higher rate of errors involving whole syllables and sub-constituents inside the syllable rime. These differences can be accounted for by recognizing the primacy of the syllable and mora in encoding Cantonese words, following proposals that have been made for Mandarin Chinese and Japanese.

## 1 Introduction

### 1.1 Motivation

There are very few large collections of spontaneous speech errors, and existing collections are heavily skewed toward Indo-European languages and other major languages of the world. In a survey designed to investigate this bias (see Appendix A), 42 (67%) of 63 corpus studies were from well-known Indo-European languages, and almost all studies were from majority languages with large populations and socio-economic power, such as English, German, and Mandarin Chinese. The overall size of these corpora in terms of the number of data points is also skewed: across all studies, only seven studies have 3,500 speech errors or greater, and all of these are from major Indo-European languages (i.e., English, Spanish, and German). As with multi-study analysis, corpus size is tremendously important because small scale studies often lack the statistical power to establish broad generalizations (Stemberger, 1982/1985). A 2011 survey found that roughly 85% of all areas of psycholinguistic research was conducted on just 10 majority languages (Anand et al., 2011). Our more focused survey yielded similar results: 84% of all speech error studies came from Anand et al.’s top 10 majority languages, and by summing the data points by language, it accounted for 94% of all of the data collected. Clearly, current speech error research does not reflect the actual linguistic diversity attested in human language.

The lack of diversity leads to two important problems. First, the lack of large data sets from a diverse set of languages means that we cannot have as much confidence as we would like to have in the speech error patterns that have been used to motivate language production models (J.-Y. Chen et al., 2002; Griffin & Crew, 2012). Second, we simply do not know what we do not know about language production processes in under-studied languages. That is, under-studied languages have unique linguistic structures and frequency distributions that present new opportunities to formulate and test hypotheses about language production processes (Jaeger & Norcliffe, 2009; Wagers et al., 2015). By excluding them from our research, we miss an opportunity to make new discoveries about language production. This article addresses the lack of linguistic diversity by introducing and exploring the sub-lexical errors of an under-studied language, Cantonese.

### 1.2 Cross-linguistic patterns

Despite the lack of linguistic diversity, literature on the available languages has developed a core set of psycholinguistic effects that have come to characterize the structure of sub-lexical speech errors (Berg, 1987; Dell et al., 1993; Goldrick, 2002; Pérez et al., 2007; Shattuck-Hufnagel, 1983; Stemberger, 1983; Vousden et al., 2000; Wells-Jensen, 2007). Sub-lexical errors include both sound and morphological errors, but we focus on sound errors here because they represent an interconnected set of data patterns, and these patterns are well-represented in our data. These cross-linguistic patterns, summarized in Table 1, have greatly informed our understanding of phonological encoding, or form retrieval processes in language production (Dell, 1986; Levelt et al., 1999; Stemberger, 1982/1985; Vousden et al., 2000).

**Table 1.** Psycholinguistic Effects Affecting Sound Errors.

a. Single phoneme effect (Nooteboom, 1969; Shattuck-Hufnagel, 1983): the large majority of sound errors are single phonemes, not sequences or features
b. Similarity effect (MacKay, 1970; Shattuck-Hufnagel & Klatt, 1979): intended and intruder sounds tend to be phonologically and phonetically similar
c. Repeated phoneme effect (Dell, 1984): intruder (error) sounds tend to share a context in intended and source words
d. Word-onset effect (Shattuck-Hufnagel, 1987; Wilshire, 1998): sound errors occur more often in word-initial position than non-initial positions
e. Phonological regularity effect (Stemberger, 1983; Wells, 1951): sound errors are phonologically regular in that they tend to obey language particular phonotactic constraints
f. Onset bias (Berg, 1991): consonantal sound errors are more common in onset position than coda position
g. Consonant bias (Nooteboom, 1969; Shattuck-Hufnagel, 1986): sound errors involve consonants more often than vowels
h. Syllable position constraint (Boomer & Laver, 1968; Fromkin, 1971): intruder (error) sounds tend to assume the same syllabic roles as they do in source words
i. Consonant–vowel constraint (MacKay, 1970; Stemberger, 1983): consonants substitute for consonants, and vowels for vowels, but consonants and vowels rarely interact

In the accounts given thus far, many of these patterns seem to be universal in the sense that they have been found, to varying degree, in every language in which they have been seriously studied. For example, the syllable context constraint is cross-linguistically robust in that it has been found in multiple studies of distinct languages, including English (Fromkin, 1971; Shattuck-Hufnagel, 1979; Stemberger, 1983; Vousden et al., 2000), Dutch (Nooteboom, 1969), German (Berg, 1991), Spanish (Berg, 1991), Swedish (Söderpalm, 1979), and Finnish (Hokkanen, 2001). Similar findings support the similarity effect and the consonant–vowel constraint: they have been documented in several studies, and no negative results have been found to date.

Other speech error patterns, while common cross-linguistically, may not be true of every language. The word-onset effect is such a pattern. Somewhere between 50% and 90% of all errors occur word-initially, which, considering the range of other positions that sound errors can occur in, is clearly above chance levels (for review, see Wilshire, 1998). However, speech errors in Spanish are argued in the work of Berg (1991), based on data from García-Albea et al. (1989), to lack a word-onset effect. Likewise, while most speech error studies document a single phoneme effect, studies of Mandarin Chinese have argued for the importance of syllables in phonological encoding on the basis that syllables seem to slip at a higher rate in these languages than other well-known Indo-European languages (J.-Y. Chen, 2000; Qu et al., 2020). Finally, some effects, like the repeated phoneme effect, have only been investigated in laboratory settings, and so they require further examination in naturalistic studies to establish that they are attested in diverse languages.

To summarize, prior research has uncovered a range of asymmetrical speech error patterns. However, many of the speech error patterns discussed above seem to require stronger validation, either because they have not been investigated in multiple studies with large baselines, or because the positive results have tended to come from a small number of related languages. Furthermore, many of the early corpus studies that these patterns are derived from suffer from methodological problems that may have skewed the results (Bock, 1996; Ferber, 1995; Pérez et al., 2007), including transcription error, missed, or misheard data, and skewed patterns due to perceptual biases, leading to further skepticism. The current study is designed to revisit this stock of psycholinguistic effects in an attempt to validate them with data from Cantonese, a language with approximately 40 million speakers that is typologically distinct from the major Indo-European languages. If the patterns are also found in a relatively large data set from an unrelated language, we can have more confidence in these key speech error patterns. Though this is the first major study of speech errors in Cantonese, it is historically related and typologically similar to Mandarin Chinese. Consequently, the data we examine will give us another representative from the Sino-Tibetan language family and support comparisons with prior research on Mandarin.

### 1.3 Accounting for cross-linguistic trends and differences

What mechanisms have been proposed to account for cross-linguistic differences in speech error patterns? A review of these mechanisms both helps us structure our investigation of Cantonese and also understand better what is at stake for theoretical models developed to account for cross-linguistic differences.

A rather important source of cross-linguistic differences is the methods used to collect and analyze speech errors. The methods used in corpus studies differ in a variety of ways, including the level of training and number of data collectors (sometimes with large numbers of untrained collectors), whether data are collected in observations from on-the-spot conversations or audio recordings, the treatment of ambiguous errors, classification systems, and general procedures for verifying collected data (see Alderete & Davies, 2019; Bock, 1996; Ferber, 1995). As a result, corpus studies differ greatly in data reliability and quality. As an example, Alderete and Davies (2019) show that differences in methodology can result in large differences in the frequency of exchange errors, like *heft-lemisphere* (for *left-hemisphere*). Exchanges are exceedingly rare in some studies, occurring in less than 1% of all errors, but they can constitute more than a third of all of the data in other studies. It seems very likely, therefore, that methodology affects both the existence of an effect and the degree to which it is found (Pérez et al., 2007; Wells-Jensen, 2007), and should therefore be a factor in assessing differences across studies.

These methodological matters aside, another clear factor in cross-linguistic differences is the fact that languages simply have different linguistic inventories. Differences in the size of phonological inventories may affect patterns in sound errors because mis-selections are more likely in large inventories (e.g., selecting a consonant out of an inventory of 10 rather than 30 consonants), and inventories may also relate to the consonant bias, since consonant inventories are in general larger than vowel inventories (J.-Y. Chen, 1999; Wells-Jensen, 2007). Languages also seem to differ in the processing of native prosodic structure. For example, Japanese differs from English in that the subparts of a VV sequence (i.e., either a long vowel or a diphthong) can be split up in speech errors, as in /koozui tjuuihoo/ → *koozuu tjuuihoo* “flood warning” (Kubozono, 1985). Kubozono (1989) argues that segments may be mis-selected in the slots defined by moras in Japanese, a phenomenon that does not exist in English. Languages may also differ in the distributions of segments in particular positions, and this may have an impact on the shape of sound errors (Lee & Goldrick, 2008). For example, the transitional probabilities from initial segments to non-initial segments differ in English and Spanish, and this may account for the relative stability of word onsets in Spanish (Griffin & Crew, 2012). The impact of language particular structures on speech error patterns has been investigated in a range of additional structures, including the impact of syllabic role on glides (Shattuck-Hufnagel, 1986; Shen, 1993; Wan, 1997), tone structure (Alderete et al., 2019; J.-Y. Chen, 1999; Gandour, 1977; Wan & Jaeger, 1998), language particular morphology (Abd-El-Jawad & Abu-Salim, 1987; Berg & Abd-El-Jawad, 1996; Han et al., 2019), stress patterns (Berg, 1991; Ohala & Ohala, 1988; Shattuck-Hufnagel, 1986), skeletal CV structure (Hartsuiker, 2002; Stemberger, 1984), and syllable inventories (J.-Y. Chen, 2000; T.-M. Chen et al., 2004).

In terms of their theoretical implications, these cross-linguistic differences due to language particular structure do not necessary entail major differences in the underlying processor. For example, if a language has many more consonants than another, or glides are part of the syllable onset in one language, and the nucleus in another, these languages can use the same underlying processor and observed differences can arise from the differences in linguistic structure. For example, diphthongs may be represented as indivisible wholes in the lexical network of English, but as combinations of individual vowels in Japanese, accounting for the fact that the sub-parts of diphthongs can be mis-selected in Japanese but not English (Kubozono, 1989). Indeed, Wells-Jensen (2007) examines speech error patterns from five languages with varying degrees of linguistic complexity, and argues that the differences among these languages do not warrant major differences in the underlying processing model.

Some cross-linguistic differences, however, have in fact been claimed to arise from differences in processing. The processing of syllables seems to differ in Chinese languages with small syllabaries relative to languages with much larger syllable inventories, like English and Dutch. Syllables appear to slip at a much higher rate in Chinese languages (J.-Y. Chen, 2000), and they have been argued to be central units of production planning in Mandarin because of their impact on naming latencies in form preparation experiments (J.-Y. Chen et al., 2002). The so-called proximate unit hypothesis states that languages differ in the planning units that immediately follow the selection of lemmas (O’Seaghdha et al., 2010). In languages like English, segments are the proximate unit and are selected immediately after lemma activation, whereas in Mandarin, where syllables play a more central role, syllables are the proximate unit. Whether or not these differences in the processing model can emerge from the attested patterns and general processes of language acquisition is an open question (J.-Y. Chen & Dell, 2006; T.-M. Chen et al., 2004), but it is clear that these theoretical innovations involve more than differences in the make-up of phonological inventories and their frequency distributions.

The present study examines 2,245 sub-lexical speech errors from Cantonese with these issues in mind. Cantonese has a syllable template similar to Mandarin, and, while its syllable inventory is not quite as reduced as Mandarin (Duanmu, 2007), it has a much smaller inventory than well-known Indo-European languages. Together with other structural differences in the structure of the vowel system, the syllabification of consonants, and general morphological differences, Cantonese provides a fresh test of the standard stock of psycholinguistic effects listed in Table 1. By testing Cantonese data against these effects, we have a new opportunity to validate them, and in doing so, support the models intended to account for them. We will also investigate new hypotheses about phonological encoding made possible by the unique structures of Cantonese, including the encoding of whole syllables and the interchangeability of syllable-internal constituents in the rime. Finally, we will return to the larger question of whether universal models of language production processes are robust to these cross-linguistic differences, or whether they require assumptions about the processing of phonological structure that go beyond mere differences in linguistic inventories.

## 2 Methods

### 2.1 Cantonese sound structure

The majority of speech errors investigated below operate on Cantonese phonological structures, and these errors are shaped by Cantonese segmental and prosodic phonology. A summary of these structures and phonological system is therefore necessary to interpret the results below.

The sound inventory of Cantonese includes 19 consonant phonemes and 19 vowel phonemes. The consonant phonemes broken down by manner class are stop sounds (*p, pʰ, t, tʰ, k, kʰ, kʷ, kʷʰ*), fricatives (*f, s, h*), affricates (*ts, tsʰ*), nasals (*m, n, ŋ*), and approximants (*l, w, j*). Eight of the vowel phonemes are monophthongs (*i, e, y, œ, ɐ, aː, o, u*), and the remaining 11 vowel phonemes (*ei, œi, ɐi, aːi, oi, ui, iu, eu, ɐu, aːu, ou*) are diphthongs formed by combining one of the monophthongal vowels with a high vowel as the second component. Our transcription here and throughout is phonemic and uses symbols from the International Phonetic Alphabet. Cantonese vowels exhibit considerable allophonic variation (Bauer & Benedict, 1997), but, for the most part, our investigations below do not delve into this level of analysis.

These phonemes are organized into a (C) X_1_(X_2_) syllable template, which can be broken down further into the onset C and rime X_1_(X_2_). The onset C slot can be filled by any of the 19 consonants, or left empty in onsetless syllables. Open syllables can either take the shape (C)X_1_, filling X_1_ with any of the monophthongal vowels except *ɐ* (which must occur in closed syllables), or (C) X_1_X_2_ filled with a diphthong. Closed syllables, on the other hand, involve filling X_1_ with a monophthong and X_2_ with a nasal or voiceless stop. Consonants filling this X_2_ slot are called coda consonants. In a small number of morphemes, syllables can also be composed of just a syllabic nasal, *m̩* or *ŋ̩*. In addition to the constraints embodied by the syllable template, consonant and vowel combinations are restricted by a set of phonotactic constraints. These include constraints that target the place of articulation of onset and syllable-final consonants and prohibit specific CX_1_, X_1_C and CX_1_C structures (Cheung, 1986; Yip, 1997). There is a parity between consonants and vowels in the sense that both can occupy the X_2_ position, which can either be analyzed as X slots in a syllable template or by recognizing the mora in Cantonese.

### 2.2 The corpus: SFUSED Cantonese 1.0

The Open Science Framework (OSF) project page (https://osf.io/er8v7/) provides access to all of the sub-lexical data, including the long-form records of all the sub-lexical errors discussed here. SFUSED stands for Simon Fraser University Speech Error Database, and SFUSED Cantonese 1.0 refers to the current version of the Cantonese language data set in this database (Alderete & Chan, 2018). The methods for the larger SFUSED project are explained and analyzed in Alderete and Davies (2019). Here, we give a crisp overview of the methods of constructing this database, with a focus on sub-lexical speech errors.

In this project, speech errors are defined as “an unintended, nonhabitual deviation from a speech plan” (Dell, 1986, p. 284), following standard practice in the field. This definition includes sound and word errors of the various types discussed in Section 2.3, but excludes false starts, idiolectal or dialectal variants, changes to a speech plan, and established patterns of variation, like the patterns that result from casual speech phonology in Cantonese (Bauer & Benedict, 1997; Matthews & Yip, 2011). The corpus also includes phonetic errors (i.e., mis-articulations of correctly selected sounds) that are distinct from phonological errors (i.e., mis-selections of discrete sound categories) because of their increasing importance in speech analysis (Frisch & Wright, 2002; Goldrick & Blumstein, 2006), but they are not discussed here.

Speech errors were collected from roughly 32 hours of audio recordings by a team of four trained data collectors. The recordings came from three different podcast series in which commentators discuss a variety of topics (e.g., film and television, lifestyle, and interpersonal relationships) in unscripted conversations. Podcast series were chosen that had high production quality, a balance of speakers for age and gender, and long intervals of unscripted speech (pre-planned or read material was not used). The speech errors were produced by 21 different native speakers, with 17 of these producing 50 or more errors.

The data collectors were native speakers of Cantonese who were also fluent, or in one case, semi-fluent, in English. Three data collectors were advanced undergraduate students in Linguistics at Simon Fraser University, and these students did the bulk of the data collection. A fourth data collector was a graduate student specializing in Cantonese linguistics and psycholinguistics, and she directed the management of the database and classification of the errors. Beyond their background in linguistics, all data collectors and analysts learned how to detect and analyze speech errors by undergoing a month of training. This training involved an introduction to spontaneously produced speech errors, phonetic training in Cantonese, and three 5-hour listening exercises. In the listening exercises, data collectors were asked to detect errors in pre-screened recordings, and then given detailed feedback on correctly identified errors, missed errors, and errors that they submitted but did not meet the standard definition of a speech error. All four of the data collectors reached a high degree of accuracy and consistency in the training (for full analysis, see the SFUSED Cantonese documentation).

The larger workflow involved the following steps. Two data collectors were assigned to each audio recording, and after collecting speech errors for these recordings, the data collectors submitted their proposed errors to the database manager who then merged the entries and batch imported them into the database. The data analyst re-examined each submitted error to verify that the submitted errors were indeed errors and then she classified the error into the types explained in Section 2.3. Often, especially for borderline cases, the data analyst sought a second option before classifying or rejecting submitted errors. A total of 3,676 errors were submitted in this way, but only 2,502 of these (68.06%) were retained as true speech errors (see Alderete & Davies, 2019, on the basic patterns of submitted but rejected speech errors).

### 2.3 Classification of speech errors

Speech errors in this study are classified using a standard system of categorizing speech errors (Dell, 1986; Shattuck-Hufnagel, 1979; Stemberger, 1993), adapted to the structures of Cantonese. The SFUSED Cantonese documentation explains these assumptions about classification in rich detail, but in general they follow similar adaptations of the standard taxonomy for Chinese languages (J.-Y. Chen, 1999; Shen, 1993; Wan & Jaeger, 1998). The analysis of each error requires establishing (i) the intended sound or word, (ii) the intruder that supplants the intended (potentially the null element ∅ in the case of deletion), and (iii) source sounds or words, if they exist, that are identical to the intruder. So-called contextual errors have source elements for intruders in the linguistic context, and non-contextual errors lack them. Speech errors are further cross-classified by type (i.e., substitution, deletion, addition, shift, etc.), unit (e.g., segment, morpheme, word), and direction (perseveration, anticipation, and exchange). When more than one classification is possible (e.g., sound errors that result in actual words can also be treated as lexical substitutions), both possibilities are recorded in the database, and Occam’s razor is used to establish the superior analysis (Stemberger, 1982/1985).

### 2.4 Data analysis

In general, we are interested in testing for the psycholinguistic effects in Table 1 on the Cantonese data. Such tests typically involve documenting the frequency of a pattern in the corpus data, and then determining whether the documented pattern deviates from chance. Chance rates are calculated on a case-by-case basis, but they are determined in part by the phonological inventory (e.g., possibilities allowed by the segment inventory for mis-selection) and the frequencies of patterns in the language as a whole (e.g., how common initial slots are relative to non-initial slots). In the more straightforward cases, we use a chi-square test to probe deviation from chance (Shattuck-Hufnagel, 1979; Stemberger, 1989). However, the similarity effect is not suitable for this test, so that, it was tested using a Mantel test for correlations between two matrices (see Alderete et al., 2019).

## 3 Results

### 3.1 Overview of the patterns and data quality

We start with an overview of the distribution of error patterns (Table 2). As explained in Section 2.3, sub-lexical errors are broken down by the type of process (e.g., substitution vs. addition of a sound), unit (segments, tone, and morphemes), and the distinction between phonological processes affecting categorical sound units and other processes (phonetic and reduction) that do not. Most of the analyses below focus on phonological errors. See Appendix B for the record ID numbers to look up these and other examples below in SFUSED Cantonese 1.0.

**Table 2.** Distribution of Error Types in SFUSED Cantonese 1.0.

Error type	Counts	Illustrations
Sub-lexical errors (90.16%)		
Phonological errors		
Phonological substitution	1,152	/mai23/ → **p**ai23 “rice”
Phonological addition	116	/uk55/ → **l**uk55 “house”
Phonological deletion	88	/si22jip22/ → si22ji_22 “career”
Phonological exchange	2	/li55 ti55/ → **t**i55 **l**i55 “these”
Phonological shift	1	/tsʰœt55hœi33/ → tsʰœ**i**t55hœ33 “to go out”
Tone substitution	420	/hei33kʰek22/ → hei**23**kʰek22 “drama”
Complex set of processes	321	/jyn21tsʰyn21/ → jyn21**ts**yn**33** “completely”
Other sub-lexical errors		
Sequential blends/reductions	54	/lei23 jiu33/ → liu23 “you must”
Phonetic errors	70	/sy55/ → s**i-y**55 “book”
Morphological errors	26	/paːt33kʷaː33keŋ33/ → paːt33kʷaː33___ “feng shui mirror”
Word and phrase errors (9.84%)	252	
*Total errors*	2,502	

The methods used to collect speech errors enable us to give a detailed analysis of data quality. Using a statistical procedure known as capture–recapture (Chao, 2001), we estimate that a speech error occurred at least as frequently as every 34 seconds, or roughly, two errors a minute. Our data collectors detected 66.52% of this estimated total. While it is clear from this estimate that our data collectors missed many errors, this two-thirds coverage of the estimated total population in SFUSED Cantonese far exceeds the sample coverage of other large collections created from audio recordings (Alderete & Davies, 2019). The distributions in Table 2 also indicate a high percentage of sound errors and very low percentage of phonological exchanges, which are also strong indicators of good data quality (Pérez et al., 2007; Stemberger, 1982/1985). Finally, the sound errors of SFUSED Cantonese exhibit high rates of violations of phonotactic constraints (see Section 3.2) relative to other large speech error databases (Stemberger, 1983), which is another indication that our methodology is robust to perceptual biases that result in missed errors (Alderete & Tupper, 2018). In sum, these analyses support the claim that the data set is a good sample of the actual population of errors in the larger corpus.

### 3.2 Segmental patterns

#### 3.2.1 The single phoneme effect and the units of sound errors

The introduction reviewed a number of psychological effects affecting individual segments in speech errors. We start our investigation of these effects by examining the phonological units that make up the majority of sound errors. Table 3 gives the counts from the three major error types (including the two exchange errors as substitutions) by the intruding unit involved in each type. This table distinguishes units that constitute phonemes in the language (i.e., single consonants, single vowels, and diphthongs; see Section 2.1), sub-syllable constituents like onsets and rimes, entire syllables, and other sequences that cannot be described with these units. Though Cantonese does not have CC onsets, substitution errors occasionally insert a non-native cluster.

**Table 3.** Counts of Errors by Unit and Type.

Class	Units	Substitution	Addition	Deletion
Single phoneme	C	717 (62.13)	74 (63.79)	58 (65.91)
	V	280 (24.26)	37 (31.90)	27 (30.68)
	VV (diphthong)	39 (3.38)	0	0
Sub-syllabic	CC (onset)	8 (0.69)	0	0
	VC	47 (4.07)	1 (0.86)	0
Whole syllables	CV, CV(X)	43 (3.73)	3 (2.59)	0
Other	*sequences*	20 (1.73)	1 (0.86)	3 (3.41)
All single phonemes	C, V, VV	1,036 (89.7)	111 (95.69)	85 (96.59)
Total		1,154	116	88

Percentages by column are in parentheses.

These results provide strong support for the single phoneme effect: the vast majority of all error types involve just a single phoneme (between 89.7% and 96.59%). These patterns compare with the rate of single phoneme errors found in Dutch, which is at 89% (Nooteboom, 1969), but are higher than the 70% rate found in English (Shattuck-Hufnagel, 1983). The trend in Cantonese favoring single phonological elements is perhaps even stronger when one considers the fact that many of the multi-segment errors are in fact coherent units when syllable structure is considered. For example, whole syllables and VC structures that are syllabified as rimes make up approximately 8% of substitutions, and far outnumber other sequences that do not constitute a syllable or sub-syllabic unit. We return to the role of syllable structure in shaping speech errors below, but the principal take home message is that single phoneme errors dominate the data set.

#### 3.2.2 The similarity effect

Another psychological effect is the phonological similarity effect, or the tendency for phonological substitutions in which intended and intruder sounds are phonologically similar (MacKay, 1970; Shattuck-Hufnagel & Klatt, 1979). Tone in SFUSED Cantonese has been investigated and shown to exhibit a similarity effect (Alderete et al., 2019). Here, we use the same methods to probe similarity in confusion matrices for consonants and vowels.

Confusion matrices for consonant and vowel phonemes are given in Tables 4 and 5. The sum of the counts for consonant substitutions (639) and vowel substitutions (230) is lower than the total number of phonological substitutions (1,152) because we only analyze single phonemes, whereas the total includes substitutions of sequences, and we also exclude certain phonetic segments like *ʔ* and illegal allophones, which are not phonemes of the language (see Section 2.1). While we report here all vowel confusions, substitutions between two simple monophthongs are clearly more prevalent (boxed regions in Table 5): they account for 79.6% of the attested vowel confusions, but just 22.1% of the logically possible substitutions. Given the structural differences between simple and diphthongal vowels, and the small cell counts with diphthongs, we focus our analysis of the similarity effect on confusions with the eight simple vowels.

**Table 4.** Confusion Matrix for Intended (Rows) and Intruder Consonants (Columns).

	*p*	*pʰ*	*f*	*t*	*tʰ*	*s*	*ts*	*tsʰ*	*k*	*kʰ*	*k* ^w^	*k* ^wh^	*h*	*m*	*n*	*ŋ*	*l*	*w*	*j*	*T*
*p*		6	3	1					1					10				1		22
*pʰ*	9		2		6					3				3				3		26
*f*	1				1	1			1								1	1		6
*t*	1				11	3	22		7		1		2		2		9		2	60
*tʰ*		8	1	12		2	1	11	1	10			3		2	1	2		2	56
*s*				1	3		13	22					5	1						45
*ts*				20	2	27		17	6	2					1		2		9	86
*tsʰ*				1	5	17	10						1						2	36
*k*	1			10	1		8	2		16	9		5			7	5	2	5	71
*kʰ*		2		1	3	3	1	3	7			1	3			7				31
*k* ^w^			1				1		1			1				1	1			6
k^wh^																				0
*h*	3	5			8	1		3	1	4				1			2		1	29
*m*	7	1				1			2						4	1	4	3		23
*n*				6	1				1					6		11			3	28
*ŋ*									6	5			1		6		1	3		22
*l*	1	1		11	2				2				1	1		2		2	8	31
*w*	2		3								1		3	7			4		1	21
*j*			1	5		2	7		2				2	1	2	1	15	2		40
*T*	25	23	11	68	43	57	63	58	38	40	11	2	26	30	17	31	46	17	33	639

**Table 5.** Confusion Matrix for Intended (Rows) and Intruder Vowels (Columns); Monophthongs Are Boxed.

	*i*	*y*	*e*	*a*	*aː*	*œ*	*o*	*u*	*ei*	*œi*	*oi*	*ai*	*aːi*	*iu*	*ou*	*au*	*aːu*	*T*
*i*		14	7	2		3	1	5										32
*y*	18																	18
*e*	5			12	2	3	2				1	1						26
*a*	1		9		12	2	7											31
*aː*			2	17			1	1								2		23
*œ*	1		7	5			2											15
*o*			2	6	2	14		8	1	2		1		1		6		43
*u*	4	1		2		1	2			1								11
*ei*														1				1
*œi*			1				1											2
*oi*																1		1
*ai*			1		4	1	1								2			9
*aːi*																		0
*iu*			1				1											2
*ou*	2			1						5								8
*au*		1			2	3							1					7
*aːu*				1														1
*T*	31	16	30	46	22	27	18	14	1	8	1	2	1	2	2	9	0	230

Testing for similarity requires correlating the data in the confusion matrixes with a measure of similarity between segment pairs. Consonants and vowels were analyzed using a standard set of phonological features from Clements and Hume (1995), which was then used to calculate the natural class similarity (Frisch et al., 2004) of all segment pairs. Natural class similarity is the ratio of number of shared natural classes (i.e., phonological classes defined by the featural analysis) over all natural classes and it is a standard measure in the analysis of speech error similarity (Frisch, 1996). To illustrate, the pair /b p/ have a relatively high natural class similarity of 0.6250 because they differ only in voicing, and therefore share a number of natural classes (e.g., obstruents, stops, and labials). The similarity of /b k/ on the other hand is roughly half of this, at 0.2941, because they share neither the class of labials nor voicing.

A Mantel test (Glerean, 2014) was used to test for a correlation between the frequencies in the two confusion matrices and phoneme similarity (i.e., natural class similarity). Before applying the test, we also removed any effects of baseline phoneme frequencies by normalizing the data in the confusion matrices. The results showed a positive correlation for consonants (*r* = .4354, *p* > .0005) and simple vowels (*r* = .5339, *p* = .005; *p-*values were simulated with 5,000 permutations). As with tone substitutions in SFUSED Cantonese, segmental substitutions exhibit a similarity effect.

It is difficult to compare these results with past research because past research does not attempt to correlate confusability with similarity; instead deviations from chance are made on the basis of individual features (Shattuck-Hufnagel, 1986) or an impressionistic account is given based on how many confusions involve segment pairs that differ in just one or a small number of features (MacKay, 1970; Wells-Jensen, 2007). Nonetheless, our results compare, both qualitatively and quantitatively, with this prior research. We have documented that segments that share many features tend to slip more often than those that do not.

#### 3.2.3 The repeated phoneme effect

Another important psychological effect at the segmental level is the repeated phoneme effect, or the greater than chance probability that intruder sounds share the same phonetic context in both the intended and source words (Dell, 1984). The examples in Table 6 illustrate this effect and how it is documented in SFUSED Cantonese (following SFUSED conventions, error words have a “/” prefix and source words have a “^” prefix). The phonological substitution of /l/ → *t* in (a) occurs immediately before the vowel *i* in both the error and source words, illustrating the effect in word-initial position, where only following segments can supply an identical phoneme. In the substitution error /u/ → *o* in (b), the vowel is flanked by consonants on both sides, and so that, while we again find an identical phoneme in front of the vowel, there was also an opportunity for an identical consonant in the onset consonant before the vowel.

**Table 6.** Illustrations of Repeated Phoneme Effect (Repeated Phoneme Underlined).

a. Word-initial consonant substitution: Error opportunity only after intruder . . . tsik55hai22 li55 ti55 siu35siu35 . . . → tsik55hai22 /ti55 ^ti55 siu35siu35 . . . 即係 /呢 ^啲 小小 . . . → 即係 /啲 ^啲 小小 . . . “precisely, this little”
b. Word-medial vowel substitution: Error opportunities before and after intruder luk22 ko33 kok33sik55 → /lok22 ko33 ^kok33sik55 /六 個^角色 → /落 個^角色 “six characters”

Understanding the opportunity for repeated phonemes is important to understanding the chance probability of their occurrence. In the simple case of word-initial consonant substitutions (a), there is about a 1-in-19 chance (or a .053 probability) of a repeated phoneme: the following vowel could be one of the 19 vowel phonemes. In word-medial contexts, we need to calculate both forward and backward opportunities on a case-by-case basis, but the chance rates of a repeated phoneme are likewise very low. For example, the 1-in-19 chance (.053 probability) of an identical preceding onset together with a 1-in-8 chance (.125 probability) of an identical following consonant or vowel (which are more limited) yields a probability of .178 using the sum rule (Bod, 2003). The exact chance estimate also involves considering the phonotactic restrictions on possible vowel–consonant combinations and the precise contexts of intruder sounds, but it will certainly be skewed toward the single opportunity estimate because roughly half (81 of 166) of all cases with repeated phonemes occur word-initially or word-finally. A conservative estimate would therefore place the overall chance estimate mid-point between .053 and .178, or approximately 12%. These estimates are far higher (and therefore easier to disprove) than those given in MacKay (1970) for repeated phonemes, who made estimates based on exchanges in the Meringer corpus (Meringer & Mayer, 1895) and how word boundaries are included in the analysis (i.e., 4% chance of repeated phoneme without counting boundaries as phonemes, 8% with boundaries).

The actual distribution of repeated phonemes in speech errors is much higher, occurring at a rate of approximately 25%, as shown in Table 7 for phonological substitutions and additions. Descriptively, this rate is very close to the rate of repetition documented in the work of MacKay (1970), which was 24% (again, without counting the word boundary in the larger analysis).

**Table 7.** Repeated Phoneme Effect.

Type	No	Yes	Total
Substitutions	468 (75.73)	150 (24.27)	618
Additions	41 (71.93)	16 (28.07)	57
Total	509 (75.41)	166 (24.59)	675

Percentages by row are in parentheses

A goodness of fit test using the 12% chance estimate indicates that the rate of repeated phonemes is significantly above chance, χ^2^(1) = 100.172, *p* = 0. Given the uncertainty of the actual chance rate discussed above, we can perform the same test using the highest possible chance rate of 18%, which again is significant, χ^2^(1) = 19.94, *p* < .0005. It is clear, therefore, that however the chance rate is calculated, repeated phonemes occur in our corpus of speech errors significantly above chance.

#### 3.2.4 The word-onset effect

Another pattern affecting segments is the so-called word-onset effect, the finding that sound errors are more commonly observed in word-initial position than elsewhere (Wilshire, 1998). To investigate this in SFUSED Cantonese, we focus on phonological substitutions because their baselines are larger and the effects of context are not uniform in phonological additions and deletions. As shown in Table 8, phonological substitutions occur slightly less often word-initially than non-initially, or about a 44% rate of occurrence averaging across both contextual and non-contextual substitutions. The chance rate of a segment occurring word-initially in this same data set is 22.24% (of all 1,151 phonological substitutions in the corpus, excluding monosegmental words, 1,151 segments are initial and 4,025 are non-initial, giving a 22.24%–77.76% breakdown between initial and non-initial segments). Goodness-of-fit tests using this chance rate to calculate expected rates of initial errors (reported in Table 8) show that SFUSED Cantonese indeed exhibits a strong word-onset effect.

**Table 8.** Phonological Substitutions by Word Position.

Class	Initial	Non-initial	Total	Significance
Contextual	274 (45.74)	325 (54.26)	599	χ^2^(1) = 189.96, *p* = 0
Non-contextual	215 (42.16)	295 (57.84)	510	χ^2^(1) = 115.86, *p* = 0
Total	489 (44.09)	620 (55.91)	1,109	χ^2^(1) = 305, *p* = 0

Percentages by row are in parentheses

However, most of the initial substitutions occurred in onset position. For example, 270 of the 274 initial contextual substitutions occurred in onsets, and only four errors involved vowel substitutions in the syllable nucleus (with vowel-initial words). This asymmetry raises the question of whether the apparent word-onset effect is in fact due to a preference for errors in syllable onsets, which is attested in SFUSED Cantonese (see below) and argued to be a factor in word-initialness in other studies (Berg, 1991). To investigate this further, we drill down into the onset errors from this data set in polysyllabic words (mono-syllabic words are excluded because they do not distinguish initial vs. non-initial). As shown in Table 9, disyllabic words have a significant association with initial position, though three- and four-syllable words, for which we have limited data, do not have the same effect. The near two-thirds majority in disyllabic words is consistent with other corpus studies (Vousden et al., 2000; Wilshire, 1998), and therefore supports the contention that there is a word-onset effect that is independent of syllable-level effects. We expect the same pattern with words greater than two syllables, but do not have sufficient data to confirm this hypothesis at this time.

**Table 9.** Onset Errors by Word Position and Syllable Count.

Syllable count	Chance (initial)	Initial	Non-initial	Total	Significance
2 syllables	50%	103 (62.05)	63 (37.95)	166	χ^2^(1) = 9.163, *p* = .0025
3 syllables	33.33%	12 (41.38)	17 (58.62)	29	*Not significant*
4 syllables	25%	6 (24)	19 (76)	25	*Not significant*

Percentages by row are in parentheses

#### 3.2.5 The phonological regularity effect

Another important principle shaping sound errors is the phonological regularity effect. Sound errors in general are phonologically regular in the sense that they tend to obey phonotactic constraints, or the language particular rules for combining segments (Boomer & Laver, 1968; Garrett, 1975; Wells, 1951). The phonological regularity effect is not an absolute constraint, because prior research has shown that, while sound errors are by and large phonologically regular, they do admit phonotactic violations to a limited degree (Alderete & Tupper, 2018; Stemberger, 1983).

SFUSED Cantonese cross-classifies speech errors by regularity, coding errors that violate phonotactics and identifying them by class (see Section 2.1). The counts of errors that do not violate phonotactics (regular) versus those that do (irregular) are shown in Table 10, cross-classified by the five major error types where this distinction is meaningful. As with other speech error collections, the sound errors of SFUSED Cantonese are by and large regular (91.07%), and the rate of phonotactic violations is low (8.93%). However, this rate is rather high when compared to the findings of other studies. For example, Alderete and Tupper (2018) found that about 5.5% of the speech errors in SFUSED English had phonotactic violations, roughly 4% lower than these results.

**Table 10.** Phonological Regularity and Irregularity.

	Regular	Irregular	Violation illustrated	Total
Substitutions	1,037 (89.86)	117 (10.14)	/jat55/ → jatʰ55	1,154
Additions	90 (77.59)	26 (22.41)	/tsau22/ → ftsau22	116
Deletions	82 (93.18)	6 (6.82)	/jɐŋ21/ → lɐ21	88
Tone errors	419 (99.76)	1 (0.24)	/kam55/ → kam45	420
Complex set	279 (88.29)	37 (11.71)	/mou23/ → pom23	316
Total	1,907 (91.07)	187 (8.93)		2,094
Adjusted total	1,907 (95.11)	98 (4.89)		2,005

Percentages by row are in parentheses

One important difference between these two data sets may explain the contrast. SFUSED Cantonese has a large number of speech errors that are irregular because they contain non-native segments, as in /wan22juŋ22/ → *[v]an22juŋ22* “to utilize.” These non-native segments are usually from English, though they are clearly sound errors and they are not to be confused with code-switching where a talker switches from one language to another. The percentage of irregular errors due to non-native segments is much higher in SFUSED Cantonese (47.59%) than SFUSED English (9.65%), presumably because all of the talkers in the Cantonese conversations are bilingual in English and Cantonese. When irregular errors from non-native segments are removed (i.e., the adjusted total in Table 10), the rate of phonological regularity is 95.11%, which is more on par with the 94.5% rate found in Alderete and Tupper (2018). This difference between SFUSED Cantonese and SFUSED English raises interesting questions about how phonological representations are accessed in the bilingual mind (see Section 5). To summarize, the sound errors of SFUSED Cantonese are in general phonologically regular, obeying native phonotactics in a larger majority of the data when we factor out problematic non-native segments.

### 3.3 Syllable-level patterns

A number of speech error patterns are shaped by syllable structure. That is, the frequency with which certain patterns occur are determined by how segments are syllabified, and how the syllabic roles of sounds in the linguistic context affect phonological encoding. As with many cases above, phonological substitutions are the best data to investigate the role of syllable structure because additions and deletions have relatively small baselines that are difficult to interpret. Additions and deletions are also shaped by marked and unmarked syllable structure (Béland & Paradis, 1997; Buchwald, 2009), which further complicates analysis because the removal or addition of a segment in specific syllabic positions is influenced by syllable structure constraints, a bias that is not found in substitutions.

#### 3.3.1 Syllabic roles and effects of onset, consonant, and whole syllables

The syllabic role of the intruding sound has a significant impact on the distribution of phonological substitutions. As shown in Table 11, substitutions of consonants in the onset position are the most frequent pattern, and they are far more frequent than consonant substitutions in coda position (58.08% vs. 7.90%). The next most frequent pattern is a vowel substitution in the nucleus position, which is in turn much more frequent than substitutions involving the rime of a syllable, the entire syllable itself, or either sub-part of a VV nucleus.

**Table 11.** Phonological Substitutions by Syllabic Role.

Syllabic role	Example	Non-contextual	Contextual	Total
Onset	/ku35/ → wu35	260	372	632 (58.08)
Nucleus	/mau23/ → mœ23	99	117	216 (19.17)
V1 of Nucleus	/ai22/ → oi22	15	41	56 (4.97)
V2 of Nucleus	/jau23/ → jai23	9	7	16 (1.42)
Coda	/tak55/ → taŋ55	49	40	89 (7.90)
Rime	/kaː/ → kik55	36	17	53 (4.70)
Syllable	/tse35/ → tei35	51	14	65 (5.77)
Total		519	608	1,127

These facts support a strong bias for onset errors and a general preference for substitutions of consonants over vowels (Nooteboom, 1969; Shattuck-Hufnagel, 1986; Vousden et al., 2000). The bias for consonantal errors over vowels (i.e., errors in onset and coda positions vs. errors in the nucleus) is slightly stronger in SFUSED Cantonese (68.62%) than in the comparable data in Nooteboom’s Dutch corpus (59.15%), but they reflect the same tendency. Likewise, the onset bias here is similar to that found in the work of Vousden et al.’s (2000) study of English, which found that errors in the onset are eight times more frequent than errors in the coda: onset errors are roughly nine times more frequent in SFUSED Cantonese based on the counts given here.

Beyond these known psychological effects, there exist certain minor patterns in our data that reveal additional asymmetrical patterns of note. First, detailed studies of English have not found extensive evidence for mis-selection of the sub-components of diphthongs (Fromkin, 1971; Stemberger, 1983). However, they constitute 6.39% of all errors by syllabic role in Table 11, a non-negligible portion. Furthermore, there are roughly three times more errors in the first component of a diphthong (V1) than the second (V2). Though predicting their frequencies are difficult without a formal model, the higher rate of errors in V1 is related to two properties of this slot. First, the selection of the intended vowel in V1 (selection of one out of eight vowels) is more difficult than in V2 (one out of two, since it must be either *i* or *u*) and so we expect more errors there. Second, vowel structure in V2 is in a sense more predictable because it unfolds in real time, so that, vowel selection in V2 can be conditioned by the vowel already selected in V1 (Dell et al., 1993).

Though some of these syllabic roles seem unaffected by the linguistic context, onset errors, nucleus errors, and errors in V1 appear to be more influenced by context. That is, the number of contextual errors for these single phoneme errors in Table 11 is larger than the corresponding non-contextual errors. In general, “bigger units” like rime and syllable errors are skewed toward non-contextual errors, so that, they are less affected by context. This finding relates to Wilshire’s (1999) observation that the word-onset effect interacts with context in that it is only a property of contextual errors: the sound errors documented here at the beginning of certain domains (i.e., syllable and nucleus) are more affected by context than in other domains.

A final observation about the data in Table 11 concerns the rate of mis-selections of entire syllables. Speech errors involving the deletion, addition, or substitution of entire syllables are very rare in languages like English and Dutch (Berg, 1998; Nooteboom, 1969; Stemberger, 1983). In the Stemberger speech error corpus, there are only 13 syllable errors out of approximately 3,660 sound errors, occurring at a rate of 0.36%. Mandarin Chinese, on the other hand, appears to have a much higher rate of syllable errors. Using rather conservative methods to identify syllable errors, J.-Y. Chen (2000) found that they occur in 8.4% of his corpus. In the context of syllable encoding in Cantonese, Alderete et al. (2019) documented a rate of syllable errors somewhere between these two rates. For all sound errors, including additions, deletions, and substitutions, errors in which the error string is a coherent syllable occurred at a rate of 4.57%, which is about half of Chen’s rate for Mandarin, but still non-negligible.

While interesting, this rate, and the rate of 5.77% in Table 11, does not establish a role for syllables in phonological encoding in the sense that syllables are actively selected and can thus be mis-selected. To establish this, we require an analysis that establishes that the observed rates greatly exceed chance rates and therefore require recognizing whole syllables in word-form encoding. Following a procedure fleshed out in J.-Y. Chen (2000), expected counts of syllable errors can be predicted from the probability of simultaneous and independent slips of the sub-syllabic constituents that make up a syllable. For example, the chance rate of a CV syllable error is the joint probability of *P*(Onset) and *P*(Nucleus), the two sub-parts of a CV syllable. In Table 12, we give the expected syllable errors in SFUSED Cantonese, calculated from the error frequencies in Table 11 by syllabic role, the duration of the corpus (32 hours), and the standard assumption that words occur in spoken corpora at about 150 words a minute (Maclay & Osgood, 1959). Given these baselines, we might expect one or two CV errors and no CVC errors in our corpus. The actual frequencies, however, greatly exceed expected frequencies for both syllable types.

**Table 12.** Syllable Errors by CV Shape.

CV shape	Example	Total	Expected frequency	*P*_Error_
CV	/tsə22/ → tʰœ22	51	0.474	1.64 × 10^–6^
CVC	/kʰek22/ → haŋ22	14	0.0001	5.08 × 10^–10^

While the expected frequencies shown here may be on par with the rates observed in English (see above), they are clearly inconsistent with the actual rates of syllable errors in Cantonese. We can conclude, therefore, with J.-Y. Chen (2000) for Mandarin speech errors, that syllable errors occur at an above chance rate.

#### 3.3.2 The syllable context constraint

Another common cross-linguistic pattern in speech errors is the syllable context constraint, or the finding that intruder sounds and their source sounds from the linguistic context tend to have the same syllabic role (Boomer & Laver, 1968; Nooteboom, 1969; Shattuck-Hufnagel, 1979; Stemberger, 1983). Contextual substitution errors in SFUSED Cantonese are cross-classified by intruder and source syllabic role in Table 13, where cell counts give the frequencies of intruder/source combinations. As we can see on the diagonal, these data also exhibit a strong syllable position effect: 92% of all errors have the same syllable role in error and source words. With errors in onset position, for example, only six of the 379 similar errors (or 1.58%) came from a coda position in a source word. Furthermore, many of the mis-matched role pairings off the diagonal are actually related syllabic roles that are functionally interchangeable. For example, a vowel that can appear in the V2 position of a diphthong can also appear on its own as a nucleus.

**Table 13.** Contextual Phonological Substitutions by Syllabic Role of Intruder (Rows) and Source (Columns).

	Onset	Nucleus	Nuc V1	Nuc V2	Coda	Rime	Syllable
Onset	373				6		2
Nucleus	1	116	7	6		1	1
Nuc V1		7	35				
Nuc V2		7		5			
Coda		1			31	3	
Rime		1			1	8	
Syllable	1	3			1		6

#### 3.3.3 The consonant–vowel constraint

A final psychological effect that relates to syllable structure is the so-called consonant–vowel constraint, or the finding that consonants swap with consonants, and vowels with vowels, but consonants and vowels do not interact (Shattuck-Hufnagel, 1986; Stemberger, 1983). Interactions between consonants and vowels are extremely rare in English speech error corpora, and are attested in only a handful of examples like *Mexicl* [mɛksəkL], where the vowel *oʊ* substitutes with *l* (Shattuck-Hufnagel, 1986). As shown in Table 14, the consonant–vowel constraint is by and large respected in SFUSED Cantonese. On the diagonal, 915 of the 937 (97.65%) substitutions are within the same segment class. However, considering how rare violations of this constraint are in English, the 22 violations found here actually seem higher than what we would expect from other languages. After all, the MIT-Arizona and the Stemberger corpora had much larger baselines and only one or two violations.

**Table 14.** Consonant-Vowel Confusions (Intended Sounds in Rows, Intruder in Columns).

	Consonant	Vowel
Consonant	705	11
Vowel	11	210

An examination of these “exceptions” to the consonant–vowel constraint reveals another interesting aspect of phonological encoding in Cantonese. As shown in Table 15, the vast majority of the violations of the consonant–vowel constraint (20 of 22, or 90.9%) occur in the X_2_ slot of the Cantonese syllable, a sub-syllabic position where consonants and vowels have a kind of parity because they both freely occur in this position. The only other context for these violations, which are similar to cases like *Mexicl* in English, are rare substitutions of the syllabic nasal in the nucleus position, just like English allows syllabic sonorants to swap with vowels in rare cases.

**Table 15.** Violations of the Consonant-Vowel Constraint.

Nature of substitution	*N*	Examples
C →V in X_2_	11	/kʰek22bun35/ → kʰek22bui35
V →C in X_2_	9	/tsau22/ → tsak22
Syllabic nasal	2	/aː33/ → n33

It would seem, therefore, that the main difference between Cantonese and English is that there exists a parity among consonants and vowels in the second part of the Cantonese rime that does not exist in English. We note that while these violations of the consonant–vowel constraint are vanishingly rare in English, they are in fact attested in Japanese (Kubozono, 1989), which likewise has a formal parity between consonants and vowels in this second position of a syllable rime.

## 4 Discussion

### 4.1 Contribution to cross-linguistic trends

Our investigation of sub-lexical errors in Cantonese is motivated by a documented lack of linguistic diversity in speech error studies. Most prior studies have focused on major languages of the world, and Indo-European languages are greatly over-represented in these studies. We therefore reviewed Cantonese data against a standard stock of psycholinguistic effects that have been used to propose models of phonological encoding (Table 1). Cantonese syllable structure, consonant and vowel inventories, and supra segmentals are rather different from the major Indo-European languages that these effects have been tested against, so that, it represents a fresh test of their validity.

Perhaps the biggest contribution of the present study is the confirmation of most of these psycholinguistic effects (Table 16). Many of these effects have comparable magnitudes to the same effects in Indo-European languages: the single phoneme effect, the repeated phoneme effect, the word-onset effect, and the syllable context constraint all have parallels within 5 percentage points in past studies of Indo-European languages. Other effects reported here (e.g., the similarity effect and the onset and consonant biases), while difficult to compare directly with prior research, support qualitative conclusions on par with past studies of major languages because they both document the above chance occurrence of particular patterns. Indeed, the only effects investigated here that lead to question marks, the phonological regularity effect and the consonant–vowel constraint, seem to have plausible explanations once we dig deeper into the data. While the 95.11% regularity in SFUSED Cantonese may seem to contain too many phonotactic violations (compared to the 99% regularity of the Stemberger corpus), this rate of phonologically regular speech errors is actually directly comparable to what we have found in SFUSED English, which is arguably a more accurate characterization of phonological regularity (Alderete & Tupper, 2018). Likewise, while there are more violations of the consonant–vowel constraint than one might expect from studies of English, it turns out that these make more sense when one considers the role of the mora in phonological encoding (see below). The larger picture painted by these results is therefore that Cantonese seems to exhibit many of the same properties of speech errors documented in large corpora of English, Dutch, and German. These results can give us more confidence in these properties and the models designed to account for them.

**Table 16.** Summary of Results, Referencing Prior Tables.

Table	Psycholinguistic effect
3	Single phoneme effect: Vast majority of errors involve a single phoneme (89.7–96.59%); cf. Dutch: 89%, English: 70%
4–5	Similarity effect: Similar sounds interact more often than dissimilar sounds (correlation between confusability and similarity *r* = .4354 for consonants, *r* = .5339 for vowels); also attested in English and German
7	Repeated phoneme effect: Substitutions and additions have a greater-than-chance tendency (24.7%) to have repeated phonemes in the source words; cf. 24% in German
8–9	Word-onset effect: Substitutions occur disproportionately in word-initial positions (44% in all words, 62.05% in onsets of disyllabic words); cf. 66% rate in English
10	Phonological regularity: Phonological errors are in general phonologically regular; that is, they obey phonotactic constraints (95.11% after adjusting for non-native segments), cf. 94.5% regularity in SFUSED English
11	Onset bias: Substitutions tend to occur in onsets (58.08%); also attested in English and German
11	Consonant bias: Substitutions involving consonants (68.62%) are much more common than vowels; cf. 59.15% in Dutch
13	Syllable context constraint: In 92% of substitution errors, the intruder and source sounds have the same syllabic role; cf. 89.5% in English
14	Consonant–vowel constraint: In 97.65% of single phoneme substitutions, consonants slip with consonants, and vowels with vowels; still the 22 violations of this principle is greater than that observed in English

### 4.2 The role of the mora and the syllable in language processing

Despite the confirmation of these major patterns, there are nonetheless a number of interesting minor patterns in SFUSED Cantonese that are clearly distinct from major Indo-European languages. As stated above, there are more violations of the consonant–vowel constraint than we expect based on English. There are also mis-selections of the individual components of diphthongs, which again is not characteristic of English. Finally, Cantonese has more errors of phonological encoding involving whole syllables, which compares with Mandarin Chinese, but is unlike English and major Indo-European languages. These facts require additional theoretical assumptions about the role of syllables and the mora in phonological encoding.

**Figure 1.**
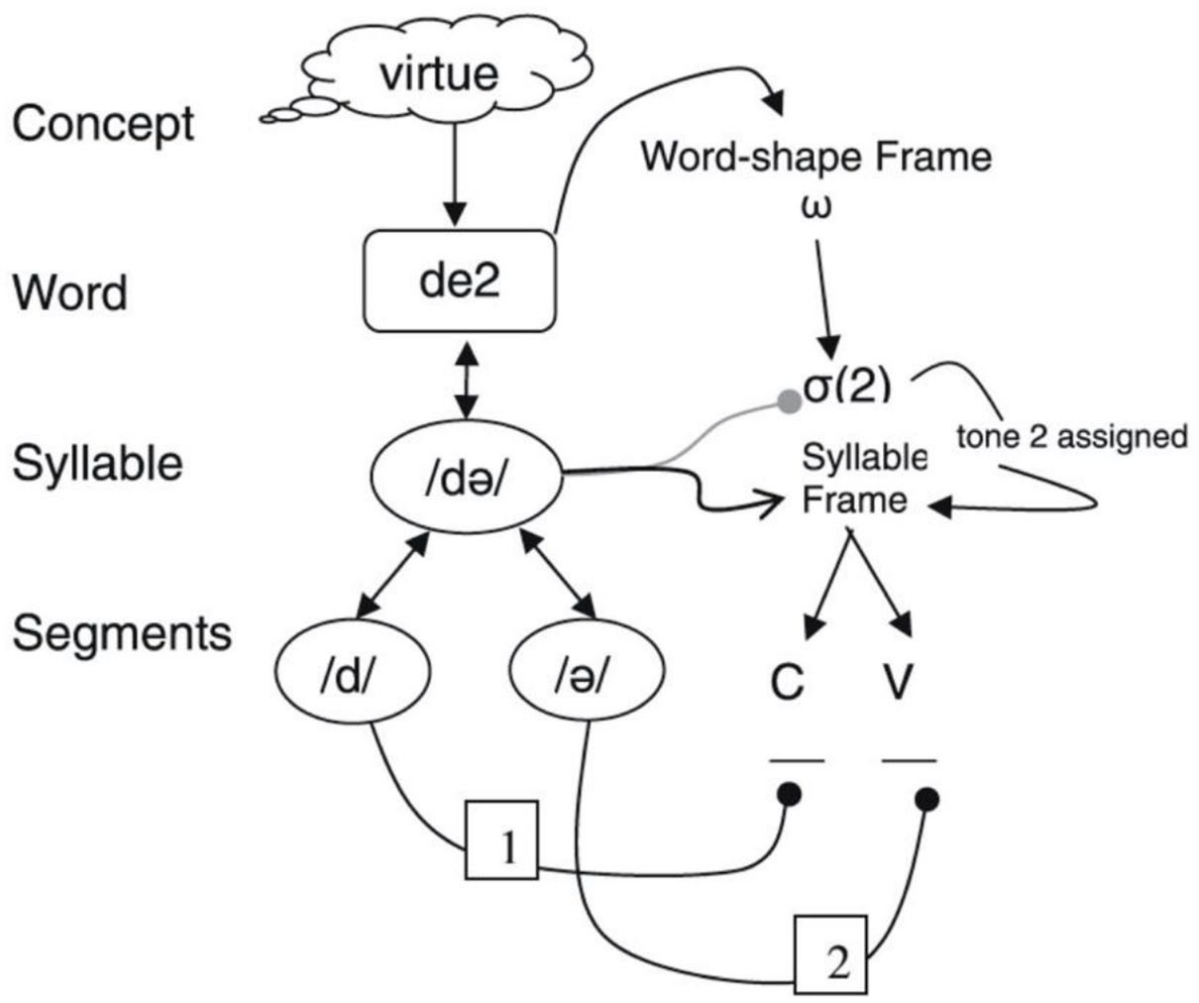
Form encoding with syllable as proximate unit in Mandarin (from O’Seaghdha et al., 2010).

The existence of whole syllable errors far above chance levels (see Section 3.3) supports assigning a privileged status to syllables in speech production planning in Cantonese. A theoretical mechanism already exists to account for the difference between English and Cantonese, namely, proximate units in phonological encoding. As sketched in Figure 1, O’Seaghdha et al. (2010) propose that languages differ in the staging of the activation of planning units, and Mandarin differs from English in that syllables are the first structures to be selected in phonological encoding (its proximate unit), directly following the activation of lemmas at the Word level. Syllable errors occur in Mandarin when syllables are mis-selected, and the segments inside this syllable are correctly selected, just as lexical errors involve mis-selected lemmas that are correctly encoded phonologically. On this account, the fact that English lacks a large number of whole syllable errors derives from the fact that segments, not syllables, are the proximate units in English, so that, syllables cannot be mis-selected. Our findings for syllable errors in Cantonese, therefore, can be seen as providing additional empirical support for the syllable as the proximate unit in a related Chinese language. This assumption is all the more convincing when one considers that we have made more conservative assumptions about the chance occurrence of syllable errors than past accounts (J.-Y. Chen, 2000).

Our findings also have implications for the encoding of rimes: the components of diphthongs can be mis-selected independently, and violations of the consonant–vowel constraint are non-negligible (Section 3.3). These patterns are uncharacteristic of English, but attested in Japanese (Kubozono, 1989). Minimally, it would seem that the slots in the Syllable Frame should reflect the syllabic template of Cantonese, that is, C X_1_ (X_2_), replacing the single V slot in Figure 1 with two X slots that range over C and V. The existence of two X slots allows the components of diphthongs to be selected separately, and also consonants to slip with vowels, and vice versa, because both segment classes can appear in the X_1_ and X_2_ slots (recall that nasals can appear in X_1_). This revision is consistent with standard representations that include syllabic roles for consonants and vowels in the lexical network (e.g., the distinction between an onset nasal [m/Onset] from the same segment that can appear in the coda [m/Coda]), because the insertion of segments into the syllable frame generally respects these syllable roles (Dell, 1986).

This accounts for the rime-internal slips in Cantonese, but does not exclude them in English. To do so, diphthongs in English must be represented in the mental lexicon essentially as wholes (as argued in Shattuck-Hufnagel, 1986). English may have a C V (C) syllable frame (see, e.g., Dell, 1988), like Cantonese, but diphthongs may not be split because they are represented as atomic wholes in the mental lexicon, and so the two components of the diphthong must be selected together. Likewise, consonants and vowels do not slip with each other because consonants are not labeled for the nucleus position, and vowels are not labeled for the coda position. Another way to formalize these ideas is to say that the X_1_ and X_2_ positions are slotted for moraic consonants, and only vowels and certain consonants are represented as moraic in the mental lexicon in Cantonese. This accords with the parallel with Japanese, which has been argued elsewhere to exhibit language processes sensitive to the mora (Otake et al., 1993). This parallel has limits, though, because the mora is an equally important phonological structure in English as well (Giegerich, 1992).

The account given above is internally consistent and successfully accounts for both the observed differences between English and Cantonese in the processing of diphthongs and the exceptions to the consonant–vowel constraint. However, an alternative to employing two processing slots exists which may be able to explain the same data. Instead of attributing errors like *ai22 → oi22* (from Table 11) to a mis-selection of V1, these could instead be analyzed as one entire diphthong (*ai*) swapping for another (*oi*), and constrained by the similarity effect (in this case, sharing the final *i*). It is difficult to gauge the predictive power of this analysis given the limits of calculating similarity of bi-partite structures and the small number of substitutions of whole diphthongs (see Section 3.2.2), but future work can investigate the potential of this analysis using experimental techniques designed specifically to assess the similarity effect (Wilshire, 1999).

### 4.3 Why do error patterns differ across languages?

These considerations lead back to larger architectural questions about the mechanisms responsible for cross-linguistic differences in the structure of speech errors. On one hand, our results seem to support the general conclusions of Wells-Jensen (2007), namely that languages do not differ substantively in their underlying language production processing, though they may have minor differences that arise from language particular structure. The confirmation of the nine psycholinguistic effects discussed above supports this view. On the other hand, syllable and sub-syllabic processing is clearly different, and potentially requires acknowledging underlying differences in production processing.

As anticipated above, methodological decisions clearly account for cross-linguistic differences. Alderete and Davies (2019) investigated a number of sub-lexical patterns, including incidence of exchange errors, phonotactics, consonant substitutions, and the word-onset effect, and found that all of them are strongly affected by methods. It is not a surprise, then, that we have found some differences, both in the existence of an effect and its magnitude, in these patterns. For example, phonotactic violations occur much more frequently in SFUSED Cantonese (roughly 5%) than in Stemberger’s corpus (1%), undoubtedly the result of the fact that, with access to audio recordings and multiple listeners, more of these difficult to detect errors can be collected. On the other hand, some effects, like the word-onset effect, are actually enhanced by perceptual biases (Pérez et al., 2007), such that, we might expect a weaker effect in studies with more robust methods. The fact that we still find such an effect in SFUSED Cantonese, with its rigorous methodology, both validates this effect and also supports the existence of a contrast with languages like Spanish (Berg, 1991). These observations are also broadly consistent with the findings of Wells-Jensen (2007), which attempted to eliminate an effect of methodology by using the same methods for five languages, and found largely uniform sub-lexical patterns.

Differences in method, however, do not account for the differences in phonotactic violations in SFUSED Cantonese and SFUSED English. As conjectured in Section 3.2, the additional 4% of phonotactic violations involving segments non-native to Cantonese, and even unattested syllable structures like complex onsets, may be due not to differences between the two languages, but instead to differences between individuals. Indeed, research on speech errors in second language acquisition has shown that many of the same psycholinguistic effects examined here are found in the speech of second language learners, and that they are affected by the structures of their first language (Poulisse, 1999). If we combine this fact with the assumption shared in many models of bilingual language production, namely that languages compete for the selection of words and sounds in the minds of bilinguals (Kroll et al., 2006; Kroll & Stewart, 1994), we expect non-native structures to “leak out” into the target language production, as we see here. Future work on cross-linguistic differences may benefit from more attention to whether the talkers of a given study are in fact bilingual, what languages they speak, and if these facts are relevant to their speech errors.

Finally, we can return to the question of whether cross-linguistic differences can largely be attributed to language particular structure, or if they seem to require different processing models. Clearly, language particular structure must be recognized. Languages have distinct prosodic systems (e.g., stress-accent vs. tone), syllabaries, and segment inventories, and these structures are distributed differently in languages. Differences in the magnitude of an effect are likely to derive from these distinctions. For example, differences in segment inventories lead to differences in the difficulty of selecting segments, as we have seen with the V1 and V2 components of diphthongs (see Section 3.3). We have also argued that Cantonese diphthongs must be represented differently than English diphthongs, namely as bi-partite structures, because they can be split in speech errors and they interact with consonants (Section 3.3). It is not clear at this time how Cantonese diphthongs come to have this structure, as contemporary analysis has analyzed them using similar devices (Giegerich, 1992; Yip, 1997). However, somehow, Cantonese vowel components come to be represented as bi-partite structures, and English diphthongs are represented as wholes.

The high rate of syllable errors, on the other hand, seems to require a model of phonological encoding for Cantonese and Mandarin that is distinct from that of English. The proximate unit (i.e., first structure activated after lemma selection) is the syllable in Mandarin and Cantonese, but it is the segment in English (O’Seaghdha et al., 2010). While it appears at first blush that these are very different models structurally, research has linked the syllable as the proximate unit to syllabary sizes in the two kinds of languages. Mandarin and Cantonese have very reduced syllabaries, relative to English. Mandarin has approximately 400 viable syllables, and Cantonese, though much higher than Mandarin with closer to 750 attested syllables (Bauer & Benedict, 1997), has a much smaller range of possible syllables than in English due to the range of consonant clusters and syllable appendices available in English. These facts raise the possibility that proximate unit effects in Chinese languages may in fact be emergent from the underlying syllabary (T.-M. Chen et al., 2004). If this is true for Cantonese, then future research can concentrate on how the facts of its reduced inventory could lead to a selection dynamics different from English, without structural differences between the two languages.

## Appendix A

### Breakdown of 63 corpus studies

To assess the bias toward majority languages in speech error research, a near exhaustive list of corpus studies was assembled and analyzed. Like the present study, the list was made up of corpus studies of speech errors from spontaneous speech; it excluded experimental studies of speech errors. The list was created by enumerating the corpus studies published in existing studies of cross-linguistic speech errors (Berg, 1987; Cutler, 1982; Poulisse, 1999) and using online search to find new studies. This step resulted in 63 corpus studies, which were then examined for the language under study, its language family, and the size of the corpus. Table A1 summarizes the findings in terms of number of studies per language and the sum total of data points (i.e., number of speech errors) from all studies of a given language. The full reference for each study, corpus size, and other details are available from the spreadsheet “63CorpusStudies” on the OSF project page.

**Table A1.** Corpus Studies by Language Family, Number, Size.

Language Family	Language	Number of studies	Size: all data points
Indo-European	English	17	27,650
	German	8	17,178
	Italian	4	4,211
	Dutch	3	3,599
	Spanish	3	12,300
	French	2	4,050
	Norwegian	2	622
	Swedish	2	882
	Welsh	1	9
Sino-Tibetan	Mandarin	6	6,606
	Taiwanese	2	2,628
Japonic	Japanese	4	5,805
Koreanic	Korean	4	3,485
Uralic	Finnish	2	2,447
	Estonian	1	150
Tai-Kadai	Thai	1	350
Afro-Asiatic	Arabic	1	911
Total:		63	92,883

## Appendix B

### Record IDs for examples

The long-form of the examples in the tables above can be looked up with the SFUSED Cantonese record ID numbers given below (from top to bottom). Table 2: 30, 338, 192, 2918, 937, 758, 17, 312, 777, 591; Table 6: 491, 1178; Table 10: 167, 148, 404, 186, 256; Table 11: 1025, 774, 16, 834, 110, 67, 696; Table 12: 2443, 764; Table 15: 708, 980, 288.

## References

[bibr1-00238309211071045] Abd-El-Jawad H. Abu-Salim I. (1987). Slips of tongue in Arabic and their theoretical implications. Languages Sciences, 9, 145–171.

[bibr2-00238309211071045] Alderete J. Chan Q. (2018). Simon Fraser University Speech Error Database—Cantonese 1.0 (First release) [www.sfu.ca/people/sfused]. Department of Linguistics, Simon Fraser University (Distributor).

[bibr3-00238309211071045] Alderete J. Chan Q. Yeung H. H. (2019). Tone slips in Cantonese: Evidence for early phonological encoding. Cognition, 191, Article 103952. 31302321 10.1016/j.cognition.2019.04.021

[bibr4-00238309211071045] Alderete J. Davies M. (2019). Investigating perceptual biases, data reliability, and data discovery in a methodology for collecting speech errors from audio recordings. Language and Speech, 62, 281–317. 29623769 10.1177/0023830918765012

[bibr5-00238309211071045] Alderete J. Tupper P. (2018). Phonological regularity, perceptual biases, and the role of grammar in speech error analysis. WIREs Cognitive Science, 9, Article e1466. 10.1002/wcs.1466 29847014

[bibr6-00238309211071045] Anand P. Chung S. Wagers M. (2011). Widening the net: Challenges for gathering linguistic data in the digital age (NSF SBE 2020: Future research in the social, behavioral, & economic sciences). https://www.nsf.gov/sbe/sbe_2020/Abstracts.pdf

[bibr7-00238309211071045] Bauer R. S. Benedict P. K. (1997). Modern Cantonese phonology (Vol. 102). Mouton de Gruyter.

[bibr8-00238309211071045] Béland R. Paradis C. (1997). Principled syllabic dissolution in a primary progressive aphasia case. Aphasiology, 11, 1171–1196.

[bibr9-00238309211071045] Berg T. (1987). A cross-linguistic comparison of slips of the tongue. Indiana University Linguistics Club.

[bibr10-00238309211071045] Berg T. (1991). Phonological processing in a syllable-timed language with pre-final stress: Evidence from Spanish speech error data. Language and Cognitive Processes, 6, 265–301.

[bibr11-00238309211071045] Berg T. (1998). Linguistic structure and change. An explanation from language processing. Oxford University Press.

[bibr12-00238309211071045] Berg T. Abd-El-Jawad H. (1996). The unfolding of suprasegmental representations: A cross-linguistic perspective. Journal of Linguistics, 32, 291–324.

[bibr13-00238309211071045] Bock K. (1996). Language production: Methods and methodologies. Psychonomic Bulletin and Review, 3, 395–421. 24213975 10.3758/BF03214545

[bibr14-00238309211071045] Bod R. (2003). Introduction to elementary probability theory and formal stochastic language theory. In Bod R. Hay J. Jannedy S. (Eds.), Probabilistic linguistics (pp. 11–37). The MIT Press.

[bibr15-00238309211071045] Boomer D. S. Laver J. D. M. (1968). Slips of the tongue. International Journal of Language and Communication Disorders, 3, 2–12. 10.3109/13682826809011435 5665921

[bibr16-00238309211071045] Buchwald A. (2009). Minimizing and optimizing structure in phonology: Evidence from aphasia. Lingua, 119, 1380–1395.

[bibr17-00238309211071045] Chao A. (2001). An overview of closed capture-recapture models. Journal of Agricultural, Biological, and Environmental Statistics, 6, 158–175.

[bibr18-00238309211071045] Chen J.-Y. (1999). The representation and processing of tone in Mandarin Chinese: Evidence from slips of the tongue. Applied Psycholinguistics, 20, 289–301.

[bibr19-00238309211071045] Chen J.-Y. (2000). Syllable errors from naturalistic slips of the tongue in Mandarin Chinese. Psychologia, 43, 15–26.

[bibr20-00238309211071045] Chen J.-Y. Chen T.-M. Dell G. S. (2002). Word-form encoding in Mandarin Chinese as assessed by the implicit priming task. Journal of Memory and Language, 46, 751–781.

[bibr21-00238309211071045] Chen J.-Y. Dell G. S. (2006). Word-form encoding in Chinese speech production. In Li P. Tan L. H. Bates E. Tzeng O. J. L. (Eds.), Handbook of East Asian psycholinguistics (Vol. 1: Chinese) (pp. 165–174). Cambridge University Press.

[bibr22-00238309211071045] Chen T.-M. Dell G. S. Chen J.-Y. (2004). A cross-linguistic study of phonological units: Syllables emerge from the statistics of Mandarin Chinese, but not from the statistics of English. Cognitive Science Society, 26, 216–220.

[bibr23-00238309211071045] Cheung K.-H. (1986). The phonology of present-day Cantonese [Doctoral dissertation, University College London, London].

[bibr24-00238309211071045] Clements G. N. Hume E. V. (1995). The internal organization of speech sounds. In Goldsmith J. A. (Ed.), The handbook of phonological theory (pp. 245–306). Blackwell.

[bibr25-00238309211071045] Cutler A. (1982). Speech errors: A classified bibliography. Indiana University Linguistics Club.

[bibr26-00238309211071045] Dell G. S. (1984). Representation of serial order in speech: Evidence from the repeated phoneme effect in speech errors. Journal of Experimental Psychology: Learning, Memory and Cognition, 10, 222–233. 6242739 10.1037//0278-7393.10.2.222

[bibr27-00238309211071045] Dell G. S. (1986). A spreading-activation theory of retrieval in sentence production. Psychological Review, 93, 283–321. 3749399

[bibr28-00238309211071045] Dell G. S. (1988). The retrieval of phonological forms in production: Tests of predictions from a connectionist model. Journal of Memory and Language, 27, 124–142.

[bibr29-00238309211071045] Dell G. S. Juliano C. Govindjee A. (1993). Structure and content in language production: A theory of frame constraints in phonological speech errors. Cognitive Science, 17, 149–195.

[bibr30-00238309211071045] Duanmu S. (2007). The phonology of standard Chinese. Oxford University Press.

[bibr31-00238309211071045] Ferber R. (1995). Reliability and validity of slip-of-the-tongue corpora: A methodological note. Linguistics, 33, 1169–1190.

[bibr32-00238309211071045] Frisch S. A. (1996). Similarity and frequency in phonology [Doctoral dissertation, Northwestern University, Evanston, IL].

[bibr33-00238309211071045] Frisch S. A. Pierrehumbert J. Broe M. B. (2004). Similarity avoidance and the OCP. Natural Language and Linguistic Theory, 22, 179–228.

[bibr34-00238309211071045] Frisch S. A. Wright R. (2002). The phonetics of phonological speech errors: An acoustic analysis of slips of the tongue. Journal of Phonetics, 30, 139–162.

[bibr35-00238309211071045] Fromkin V. (1971). The non-anomalous nature of anomalous utterances. Language, 47, 27–52.

[bibr36-00238309211071045] Gandour J. (1977). Counterfeit tones in the speech of southern Thai bidialectals. Lingua, 41, 125–143.

[bibr37-00238309211071045] García-Albea J. E. del Viso S. Igoa J. M. (1989). Movement errors and levels of processing in sentence production. Journal of Psycholinguistic Research, 18, 145–161.

[bibr38-00238309211071045] Garrett M. (1975). The analysis of sentence production. In Bower G. H. (Ed.), The psychology of learning and motivation: Advances in research and theory, Volume 9 (pp. 131–177). Academic Press.

[bibr39-00238309211071045] Giegerich H. J. (1992). English phonology: An introduction. Cambridge University Press.

[bibr40-00238309211071045] Glerean E. (2014). Mantel test—Matlab implementation. 10.6084/m9.figshare.1008724.v3

[bibr41-00238309211071045] Goldrick M. (2002). Patterns of sound, patterns in mind: Phonological regularities in speech production [Doctoral dissertation, Johns Hopkins University, Baltimore, MD].

[bibr42-00238309211071045] Goldrick M. Blumstein S. (2006). Cascading activation from phonological planning to articulatory processes: Evidence from tongue twisters. Language and Cognitive Processes, 21, 649–683.

[bibr43-00238309211071045] Griffin Z. M. Crew C. M. (2012). Research in language production. In Spivey M. J. McRae K. Joanisse M. F. (Eds.), The Cambridge handbook of psycholinguistics (pp. 409–425). Cambridge University Press.

[bibr44-00238309211071045] Han J.-I. Oh J. Kim J.-Y. (2019). Slips of tongue in the Seoul Korean Corpus of spontaneous speech. Lingua, 220, 31–42.

[bibr45-00238309211071045] Hartsuiker R. J. (2002). The addition bias in Dutch and Spanish phonological speech errors: The role of structural context. Language and Cognitive Processes, 17, 61–96.

[bibr46-00238309211071045] Hokkanen T. (2001). Slips of the tongue: Errors, repairs and a model (Studia Fennica Linguistica 10). Finnish Literature Society.

[bibr47-00238309211071045] Jaeger T. F. Norcliffe E. J. (2009). The cross-linguistic study of sentence production. Language and Linguistic Compass, 3, 866–887.

[bibr48-00238309211071045] Kroll J. F. Bobb S. C. Wodniecka Z. (2006). Language selectivity is the exception, not the rule: Arguments against a fixed locus of language selection in bilingual speech. Bilingualism: Language and Cognition, 9, 119–135.

[bibr49-00238309211071045] Kroll J. F. Stewart E. (1994). Category interference in translation and picture naming: Evidence for asymmetric connections between bilingual memory representations. Journal of Memory and Language, 33, 149–174.

[bibr50-00238309211071045] Kubozono H. (1985). Speech errors and syllable structure. Linguistics and Philology, 6, 220–243.

[bibr51-00238309211071045] Kubozono H. (1989). The mora and syllable structure in Japanese: Evidence from speech errors. Language and Speech, 32, 249–278.

[bibr52-00238309211071045] Lee Y. Goldrick M. (2008). The emergence of sub-syllabic representations. Journal of Memory and Language, 59, 155–168.

[bibr53-00238309211071045] Levelt W. J. M. Roelofs A. Meyer A. S. (1999). A theory of lexical access in speech production. Behavioral and Brain Sciences, 22, 1–75. 11301520 10.1017/s0140525x99001776

[bibr54-00238309211071045] MacKay D. G. (1970). Spoonerisms: The structure of errors in the serial order of speech. Neuropsychologia, 8, 323–350. 5522566 10.1016/0028-3932(70)90078-3

[bibr55-00238309211071045] Maclay H. Osgood C. E. (1959). Hesitation phenomena in spontaneous English speech. Word, 15, 19–44.

[bibr56-00238309211071045] Matthews S. Yip V. (2011). Cantonese: A comprehensive grammar. Routledge.

[bibr57-00238309211071045] Meringer R. Mayer C. (1895). Versprechen und Verlesen. Gbschensche Verlagsbuchhandlung.

[bibr58-00238309211071045] Nooteboom S. G. (1969). The tongue slips into patterns. In van Essen A. J. van Raad A. A. (Eds.), Leyden studies in linguistics and phonetics (pp. 114–132). Mouton.

[bibr59-00238309211071045] O’Seaghdha P. G. Chen J.-Y. Chen T.-M. (2010). Proximate units in word production: Phonological encoding begins with syllables in Mandarin Chinese but with segments in English. Cognition, 115, 282–302. 20149354 10.1016/j.cognition.2010.01.001 PMC2854551

[bibr60-00238309211071045] Ohala M. Ohala J. J. (1988). The scarcity of speech errors in Hindi. In Hyman L. M. Li C. N. (Eds.), Language, speech and mind. Studies in honour of Victoria A. Fromkin (pp. 239–253). Routledge.

[bibr61-00238309211071045] Otake T. Hatano G. Cutler A. Mehler J. (1993). Mora or syllable? Speech segmentation in Japanese. Journal of Memory and Language, 32, 258–278.

[bibr62-00238309211071045] Pérez E. Santiago J. Palma A. O’Seaghdha P. G. (2007). Perceptual bias in speech error data collection: Insights from Spanish speech errors. Journal of Psycholinguistic Research, 36, 207–235. 17186384 10.1007/s10936-006-9042-7

[bibr63-00238309211071045] Poulisse N. (1999). Slips of the tongue: Speech errors in first and second language production. John Benjamins.

[bibr64-00238309211071045] Qu Q. Feng C. Hou F. Damian M. F. (2020). Syllables and phonemes as planning units in Mandarin Chinese spoken word production: Evidence from ERPs. Neuropsychologia, 146, Article 107559. 32679134 10.1016/j.neuropsychologia.2020.107559

[bibr65-00238309211071045] Shattuck-Hufnagel S. (1979). Speech errors as evidence for a serial-ordering mechanism in sentence production. In Copper W. E. Walker E. C. T. (Eds.), Sentence processing: Psycholinguistic studies presented to Merrill Garrett (pp. 295–342). Lawrence Erlbaum.

[bibr66-00238309211071045] Shattuck-Hufnagel S. (1983). Sublexical units and suprasegmental structure in speech production planning. In MacNeilage P. F. (Ed.), The production of speech (pp. 109–136). Springer Verlag.

[bibr67-00238309211071045] Shattuck-Hufnagel S. (1986). The representation of phonological information during speech production planning: Evidence from vowel errors in spontaneous speech. Phonology Yearbook, 3, 117–149.

[bibr68-00238309211071045] Shattuck-Hufnagel S. (1987). The role of word onset consonants in speech production planning: New evidence from speech error patterns. In Keller E. Gopnik M. (Eds.), Motor and sensory processes of language (pp. 17–51). Lawrence Erlbaum.

[bibr69-00238309211071045] Shattuck-Hufnagel S. Klatt D. H. (1979). The limited use of distinctive features and markedness in speech production: Evidence from speech error data. Journal of Verbal Learning and Verbal Behavior, 18, 41–55.

[bibr70-00238309211071045] Shen J. (1993). Slips of the tongue and the syllable structure of Mandarin Chinese. In Yau S.-C. (Ed.), Essays on the Chinese language by contemporary Chinese scholars (pp. 139–161). Centre de Recherche Linguistiques sur L’Asie Orientale, École des Hautes Études en Sciences Sociales.

[bibr71-00238309211071045] Söderpalm E. (1979). Speech errors in normal and pathological speech (Travaux de L’Institute de Linguistique de Lund #14). Gleerup.

[bibr72-00238309211071045] Stemberger J. P. (1982/1985). The lexicon in a model of language production. Garland.

[bibr73-00238309211071045] Stemberger J. P. (1983). Speech errors and theoretical phonology: A review. Indiana University Linguistics Club.

[bibr74-00238309211071045] Stemberger J. P. (1984). Speech error collection and fieldwork: Some Choctaw speech errors. International Journal of American Linguistics, 50, 345–349.

[bibr75-00238309211071045] Stemberger J. P. (1985). An interactive activation model of language production. In Ellis A. W. (Ed.), Progress in psychology of language (Vol. 1, pp. 143–186). Lawrence Erlbaum.

[bibr76-00238309211071045] Stemberger J. P. (1989). Speech errors in early child language production. Journal of Memory and Language, 28, 164–188.

[bibr77-00238309211071045] Stemberger J. P. (1993). Spontaneous and evoked slips of the tongue. In Blanken G. Dittmann J. Grimm H. Marshall J. C. Wallesch C.-W. (Eds.), Linguistic disorders and pathologies. An international handbook (pp. 53–65). Walter de Gruyter.

[bibr78-00238309211071045] Vousden J. I. Brown G. D. A. Harley T. A. (2000). Serial control of phonology in speech production: A hierarchical model. Cognitive Psychology, 41, 101–175. 10968924 10.1006/cogp.2000.0739

[bibr79-00238309211071045] Wagers M. Borja M. F. Chung S. (2015). The real-time comprehension of Wh-dependencies in a Wh-agreement language. Language, 91, 109–144.

[bibr80-00238309211071045] Wan I.-P. (1997). The status of prenuclear glides in Mandarin Chinese: Evidence from speech errors. Chicago Linguistics Society, 33, 417–428.

[bibr81-00238309211071045] Wan I.-P. Jaeger J. J. (1998). Speech errors and the representation of tone in Mandarin Chinese. Phonology, 15, 417–461.

[bibr82-00238309211071045] Wells R. (1951). Predicting slips of the tongue. Yale Scientific Magazine, 3, 9–30.

[bibr83-00238309211071045] Wells-Jensen S. (2007). A cross-linguistic speech error investigation of functional complexity. Journal of Psychological Research, 36, 107–157. 10.1007/s10936-006-9036-5 17180468

[bibr84-00238309211071045] Wilshire C. E. (1998). Serial order in phonological encoding: An exploration of the “word onset effect” using laboratory-induced errors. Cognition, 68, 143–166. 9818510 10.1016/s0010-0277(98)00045-6

[bibr85-00238309211071045] Wilshire C. E. (1999). The “tongue twister” paradigm as a technique for studying phonological encoding. Language and Speech, 42, 57–82.

[bibr86-00238309211071045] Yip M. (1997). Consonant-vowel interaction in Cantonese. In Wang J. Smith N. (Eds.), Studies in Chinese phonology (pp. 251–274). Mouton de Gruyter.

